# Batch correction evaluation framework using a-priori gene-gene associations: applied to the GTEx dataset

**DOI:** 10.1186/s12859-019-2855-9

**Published:** 2019-05-28

**Authors:** Judith Somekh, Shai S Shen-Orr, Isaac S Kohane

**Affiliations:** 1 Department of Biomedical Informatics, Harvard Medical School, Boston, MA USA; 2 Faculty of Medicine, Technion – Israel Institute of Technology, Haifa, Israel; 3 Department of Information Systems, University of Haifa, Haifa, Israel

**Keywords:** Batch correction, Batch effect, Gene expression, ComBat, Principal component analysis, GTEx

## Abstract

**Background:**

Correcting a heterogeneous dataset that presents artefacts from several confounders is often an essential bioinformatics task. Attempting to remove these batch effects will result in some biologically meaningful signals being lost. Thus, a central challenge is assessing if the removal of unwanted technical variation harms the biological signal that is of interest to the researcher.

**Results:**

We describe a novel framework, B-CeF, to evaluate the effectiveness of batch correction methods and their tendency toward over or under correction. The approach is based on comparing co-expression of adjusted gene-gene pairs to a-priori knowledge of highly confident gene-gene associations based on thousands of unrelated experiments derived from an external reference. Our framework includes three steps: (1) data adjustment with the desired methods (2) calculating gene-gene co-expression measurements for adjusted datasets (3) evaluating the performance of the co-expression measurements against a gold standard. Using the framework, we evaluated five batch correction methods applied to RNA-seq data of six representative tissue datasets derived from the GTEx project.

**Conclusions:**

Our framework enables the evaluation of batch correction methods to better preserve the original biological signal. We show that using a multiple linear regression model to correct for known confounders outperforms factor analysis-based methods that estimate hidden confounders. The code is publicly available as an R package.

**Electronic supplementary material:**

The online version of this article (10.1186/s12859-019-2855-9) contains supplementary material, which is available to authorized users.

## Background

Although ultrahigh-throughput sequencing technologies for gene expression profiling that measure the expression levels of thousands of genes in a single experiment present a promising technique to discover novel biomedical phenomena, they may suffer from artifacts that can delay the discovery. The adjustment of heterogeneous gene expression data that present noise generated by a single or multiple confounding factors needs to be taken into account. Attempting to remove batch effects may result in over fitting, which results in the loss of some of the biologically meaningful components of the measurement (i.e., signal). Thus, evaluating the results of the adjustment methods is as pivotal as the batch effect removal process itself [1]. The lack of such evaluation tools may even result in an elevated distortion of the data following adjustment, introducing serious errors in the results of any downstream analysis performed. For example, a loss of an expected biological signal of healthy and diseases colorectal/breast cancer patients was detected following batch correction with PCA (principle component analysis) based method [2] and the work in [3] evaluated the extent to which various batch correction algorithms remove true biological heterogeneity using replicate samples. A pivotal challenge thus arises of how to determine whether an adjustment assists or damages the biological (i.e., non-technical) signal in the data.

Batch correction approaches can be roughly divided into three categories: (1) those aimed at removing known covariates, e.g., ComBat [4], which applies an empirical Bayes approach, (2) those aimed at removing unknown covariates, e.g., inferring hidden covariates using principal components [5] or factor analysis [6], and (3) those aimed at removing both known and unknown covariates. Several powerful approaches aimed at correcting hidden batch effects prior to differential expression analysis were suggested [7–11]. The Surrogate Variable Analysis (SVA) method [8] and its SVAseq [9] extension for RNA-seq data, used SVD (singular value decomposition) to define hidden confounders on the signal removed residual matrix. The method uses permutation tests to choose the significant singular vectors, finds a subset of genes that account for them and finally creates a surrogate vector for each gene subset. Focusing on detecting biological heterogeneity, the pSVA approach [3] reverses the common application of SVA to estimate biological heterogeneity as those features measured from genes not associated with an a-priori known technical covariates in the model matrix. The SVAPLSseq [10] method estimates hidden confounders using partial least square regression model of the original expression matrix on the primary signal removed expression matrix or using a set of control features. The RUV-2 method [11] suggested adjusting for batch effects using the variation between conditions of a-priori negative control genes known not to be altered and related to the biological factor of interest (i.e., not differentially expressed). Using factor analysis, the negative control genes were incorporated into a linear regression model to adjust for unwanted variation in a dataset resulting from batch effects. These methods are dedicated to a downstream differential expression analysis that takes into account the differential biological variation between the contrasted groups supervising their computation. This makes it less than intuitive to be utilized for the unsupervised batch correction computation required for a downstream co-expression analysis.

Recently, several combined methods were developed to account for data overcorrection. They were mostly based on assessing data variation or reducing it using factor or principle component analysis combined with prior knowledge (e.g., known batches). For example, the Harman method [12] refined principal component analyses using known batch effects to adjust for data variation related to known batches. They generated principal components on per-batch-summation of the original data. A *p*-value for the significance of the batch-related first principal component variation is then used for the data adjustment. The HCP (Hidden Covariates with Prior) method [13] also refined principal components-based analyses using known batches. To asses their method, they evaluated the accuracy of the constructed co-expression network (gene-gene pairs from the batch-corrected expression datasets) to predict functional networks based on gene ontology (GO) categories. Inferred hidden confounder factors, PEER factors [6], were used to adjust for batch effects for the GTEx human tissues-dataset [14–16]. With the aim of generating co-expression networks, [14] followed the methodology suggested in [13] to preserve the desired biological signal and used GO categories to quantify the reasonable numbers of principal components to be adjusted in each tissue with respect to the optimal GO enrichment. The work at [17] used a-priori knowledge on the true noise to evaluate adjustment methods. They used control data of technical replicates (comparing their correlation before and after batch adjustments) and principal component analysis on simulated data.

Here we present B-CeF (**B**atch **C**orrection **E**valuation **F**ramework), a novel framework for assessment of batch correction approaches on actual data considering the genuine biological signal left. Focusing on the desired downstream co-expression analysis following the batch correction, we suggest computing a metric that compares the biological signal left in the adjusted datasets, represented by gene-gene co-expression, to an a-priori external knowledgebase, a gold standard, of a genuine biological signal. The gold standard, derived from the GIANT database [18], is represented by a set of actual high confident gene-gene associations based on co-expression and protein-interaction networks derived from thousands of experiments. We use the B-CeF methodology to evaluate five batch correction methodologies applied to six representative tissues from the GTEx dataset [15, 16].

## Results

**Fig. 1:**
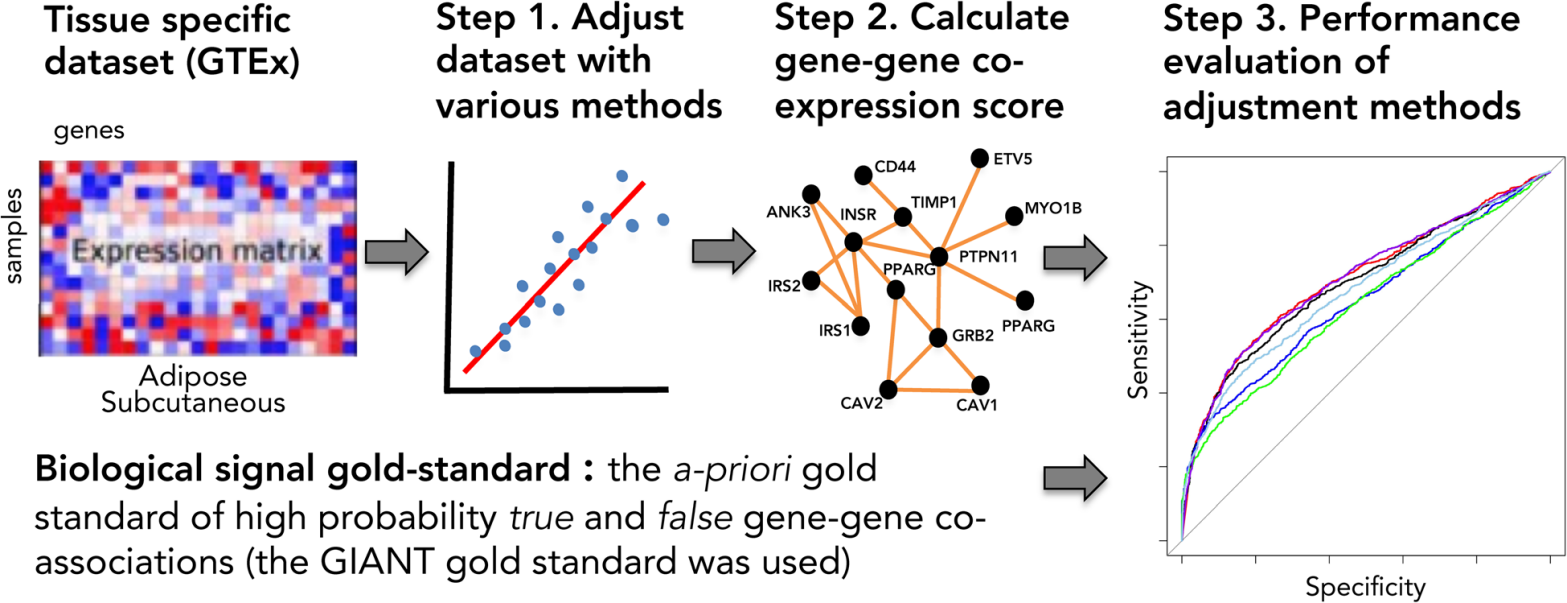
Schematic view of the framework

The B-CeF assessment framework uses a-priori gene-gene *true* and *false* associations to evaluate the effectiveness of batch correction methods to preserve meaningful biological signals (see Fig. 1 for schematic overview). A *true* gene-gene association is defined as two genes that are verified to be co-associated across multiple biological conditions (i.e., based on co-expression and biological interactions, see Methods), and *false* association is defined as two genes that are thought to not be associated. An adjustment method is considered as being effective if the number of true positive or true negative pairs in the adjusted dataset increases with respect to raw unadjusted data. Specifically, the steps of our methodology include: (1) construct the a-priori gold standard of high probability true and false gene-gene pairs (co-associations); (2) construct for the adjusted dataset a corresponding set of gene-gene pairs and their correlation coefficients and *p*-values estimation, and finally (3) evaluate the performance of each adjustment method using these p-values as scores against the gold standard pairs for generating ROC curves and AUC (see Methods). We demonstrate the B-CeF methodology by contrasting five batch correction methods and raw data.

**Fig. 2:**
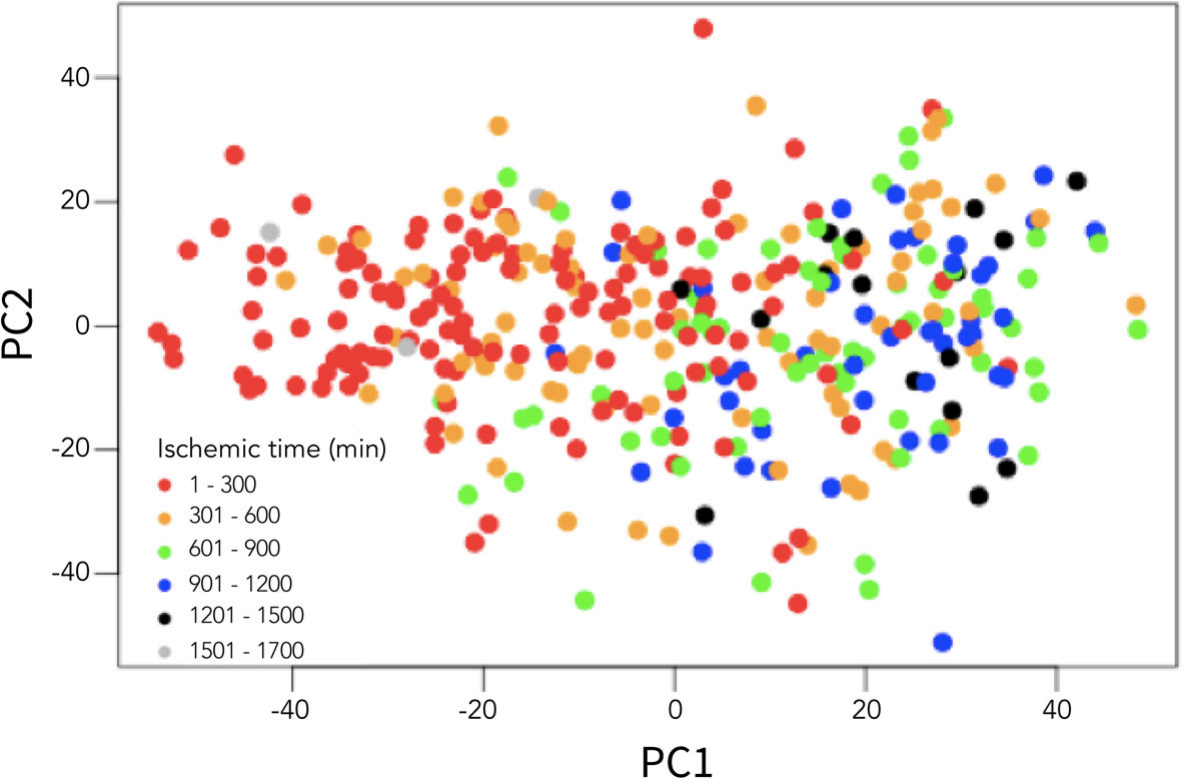
Variability related to ischemic time in the GTEx Adipose Subcutaneous dataset. Samples are shown in the PC space of the first two principal components. It can be seen that ischemic time affects the variability of the samples

GTEx was shown (see Fig. 2, [16]) to be a highly heterogeneous dataset affected by several batch effects, e.g., ischemic time, experimental batch and death type.

**Fig. 3:**
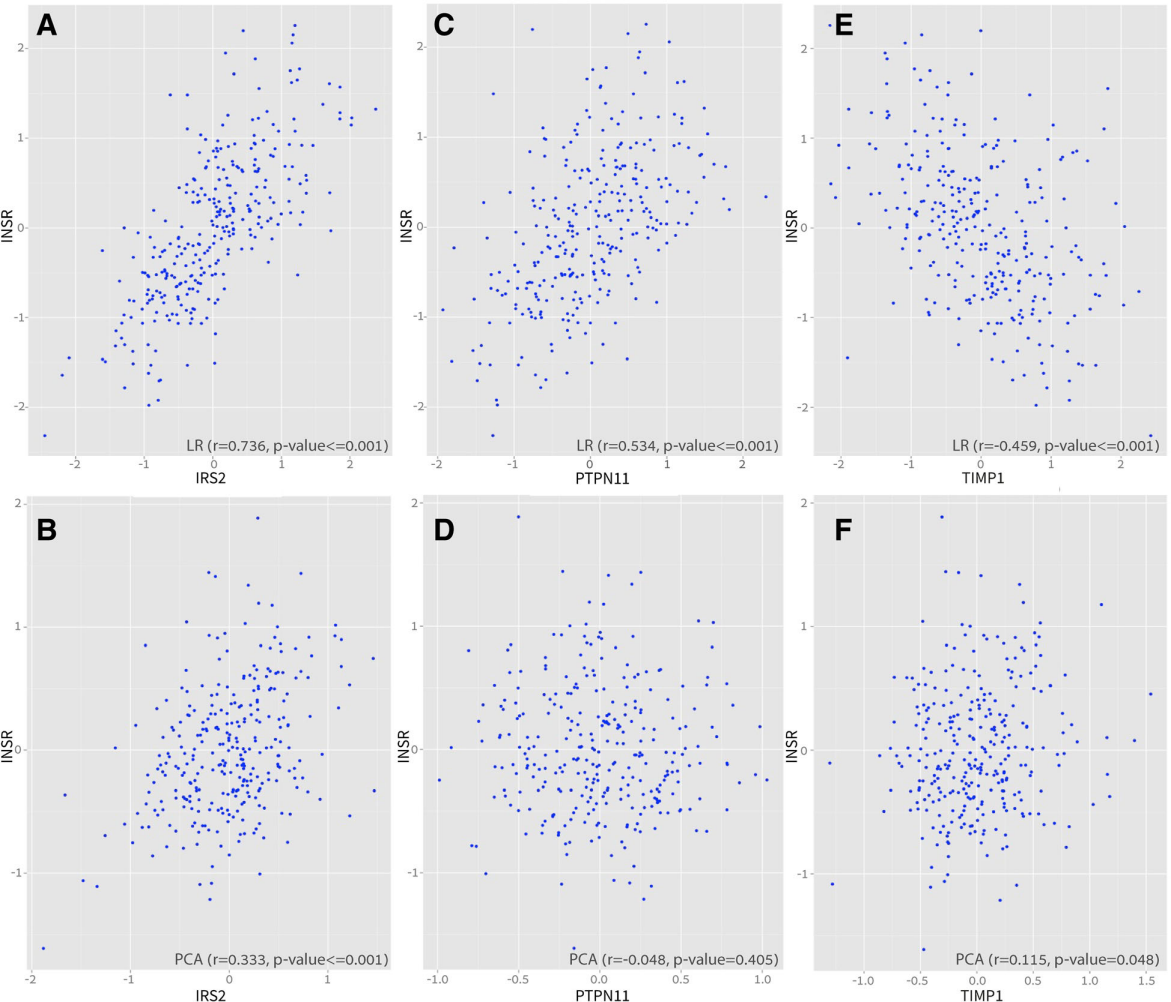
Examples of Spearman correlation coefficients of three *true* gene-gene associations. These are calculated following LR-based adjustment (using the linear regression model for known confounders) and principal components-based adjustment of the GTEx Adipose Subcutaneous dataset. The example genes are derived from the insulin signaling mechanism. **a**, **b** Example of co-expression plot of LR and PCA-based adjustment for INSR-IRS2 association. **c**, **d** Example of co-expression plot of LR and PCA-based adjustment for INSR-PTPN11 association. **e**, **f** Example of co-expression plot of LR and PCA-based adjustment for INSR-TIMP1 association. The y-axis presents the INSR (insulin receptor) expression for each sample (depicted by circles) and the x-axis the expression values of the relevant gene. Spearman correlation coefficients and *p*-values are presented at the top of each plot. It can be seen that removing most of the variability using principal components-based adjustment (PCA) can result in eliminating a desired biological signal, e.g., PTPN11 has a significant correlation coefficient to INSR (r = 0.534, *p*-value < 0.001) following the LR adjustment and non-significant (r = ~ 0, *p*-value = 0.405) following the PCA adjustment

Figure 2 shows a plot of the first and second principle component values for the Adipose Subcutaneous dataset, colored by discretized ischemic time. Ischemic time is the time in minutes that elapsed between death time and samples extraction. It can be seen that ischemic time affects the variability of the gene expression values of the samples. Figure 3 exemplifies the co-expression of three *true* co-expressed gene-gene pairs derived from the insulin-signaling pathway, INSR with IRS2, TIMP1 and PTPN11. The corresponding confidence values (the probability for the association) derived from the GIANT project [18] for these *true* associations are IRS2-INSR confidence = 0.50, INSR-TIMP1 confidence = 0.69, INSR-PTPN11 confidence = 0.69.

The biological roles of these genes are as follows. INSR [19] is a receptor tyrosine kinase, which activates the insulin-signaling pathway when bound to insulin or other ligands. INSR stimulation leads to the phosphorylation of several intracellular substrates, including insulin receptor substrates (such as IRS2). The IRS2 gene encodes the insulin receptor substrate 2, which is a cytoplasmic signaling molecule that mediates between diverse receptor tyrosine kinases (e.g., INSR) and downstream effectors. Each of these phosphorylated insulin receptor substrates serve as docking proteins for other signaling proteins, including the SHP2 (PTPN11) molecule [19]. The TIMP1 (TIMP Metallopeptidase Inhibitor 1) gene participates in the inhibition of the insulin signaling mechanism and its product levels were shown to increase as a result of hyperinsulinemia [20].

Figure 3 presents the co-expressions plots, correlation coefficients and *p*-values after adjustment with LR and PCA (see Methods). The PCA correction (principal component based correction) eliminates the expected biological signal between these biologically-related genes when compared to the LR correction (linear regression based correction of known confounders). The figure demonstrates that the correlation coefficients of INSR with the described three genes are significantly reduced following the PCA-based adjustment compared to the LR-based adjustment. For example, Fig. 3c, d show that the LR adjustment results in a significant correlation coefficient (r = 0.534, *p*-value < 0.001) for the pair = (INSR, PTPN11) as opposed to the PCA-based adjustment (r~ = 0, *p*-value > 0.1).

**Fig. 4:**
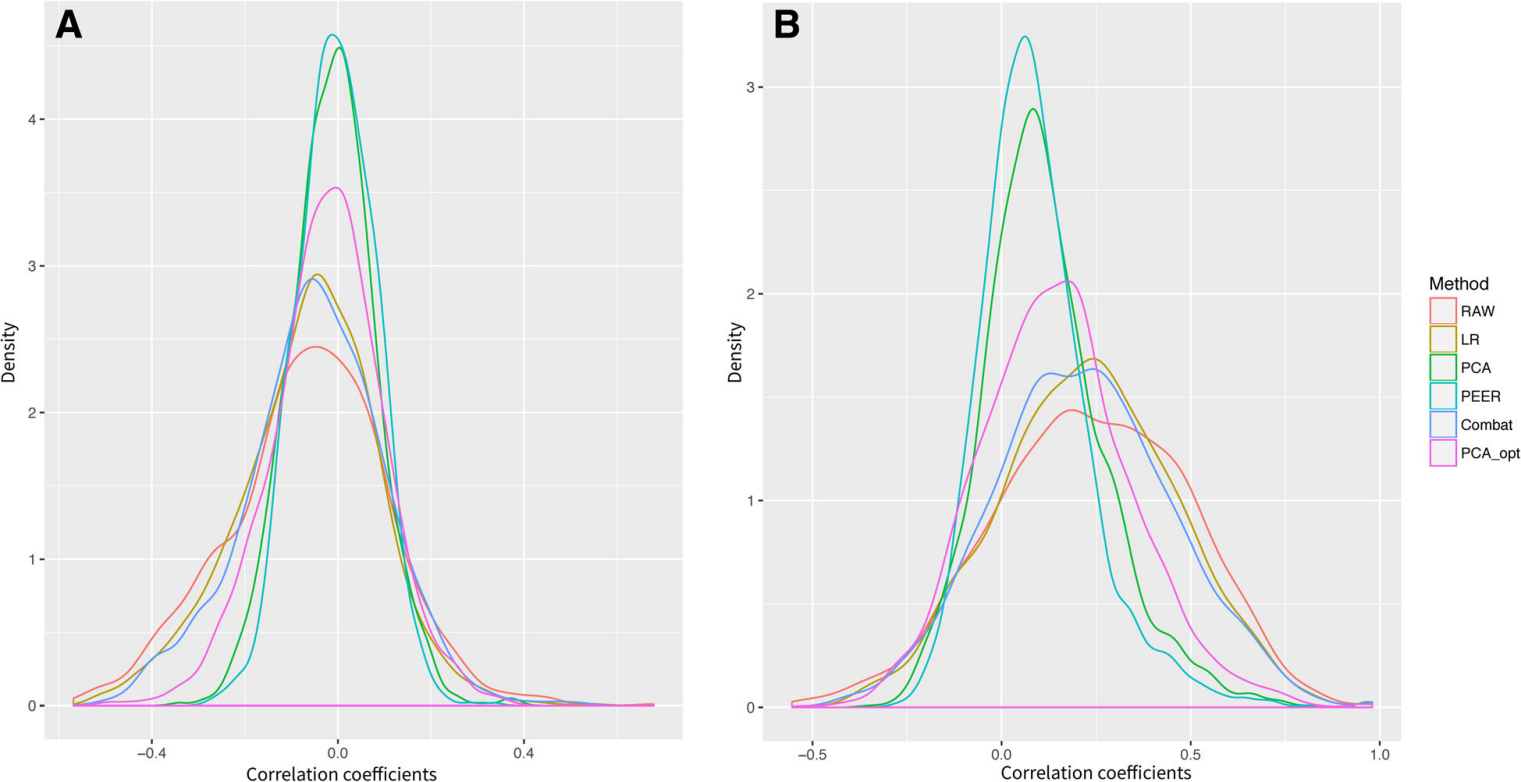
Density plots of Spearman’s correlation coefficients (r_s_) of gene-gene pairs following adjustment with five methods. (**a**) Density plot for r_s_ of *false* gene-gene pairs. (**b**) Density plot for r_s_ of *true* gene-gene pairs. We used 1796 *true* and 1179 *false* edges following adjustment with five methods and raw data for the GTEx Adipose Subcutaneous dataset. Data adjustment with hidden confounders, i.e., PEER and PCA-based covariates (colored in green and blue respectively) demonstrates a tendency toward zero mean of the correlation coefficients in the *false* and in the *true* gene-gene pairs

In the first step of our methodology we generated a high confidence gold standard of gene-gene co-associations for representing an actual biological signal. We then derived the strongest/weakest *true* and *false* gene-gene pairs (see Methods). In addition, we adjusted six tissue-specific datasets with five batch correction methods each. In the second step of the methodology we generated the co-expression networks, i.e., a gene-gene co-expression score based on correlation coefficients *p*-values (see Methods), per each adjusted dataset and tissue. Figure 4 shows the density plots of correlation coefficient values of the a-priori *true* and *false* gene-gene pairs following adjustments with five methods for the Adipose Subcutaneous GTEx dataset. A tendency toward zero mean of correlation coefficients in both *true* and *false* gene-gene pairs can be seen for data adjusted with hidden confounders that removes most of the data variability, such as using PEER or principle components (PCA). The methods that consider known confounders better preserve the expected correlations for *true* gene-gene signals. The same trend is exemplified for other tissues (see Additional file 1: Figure S5).

**Fig. 5:**
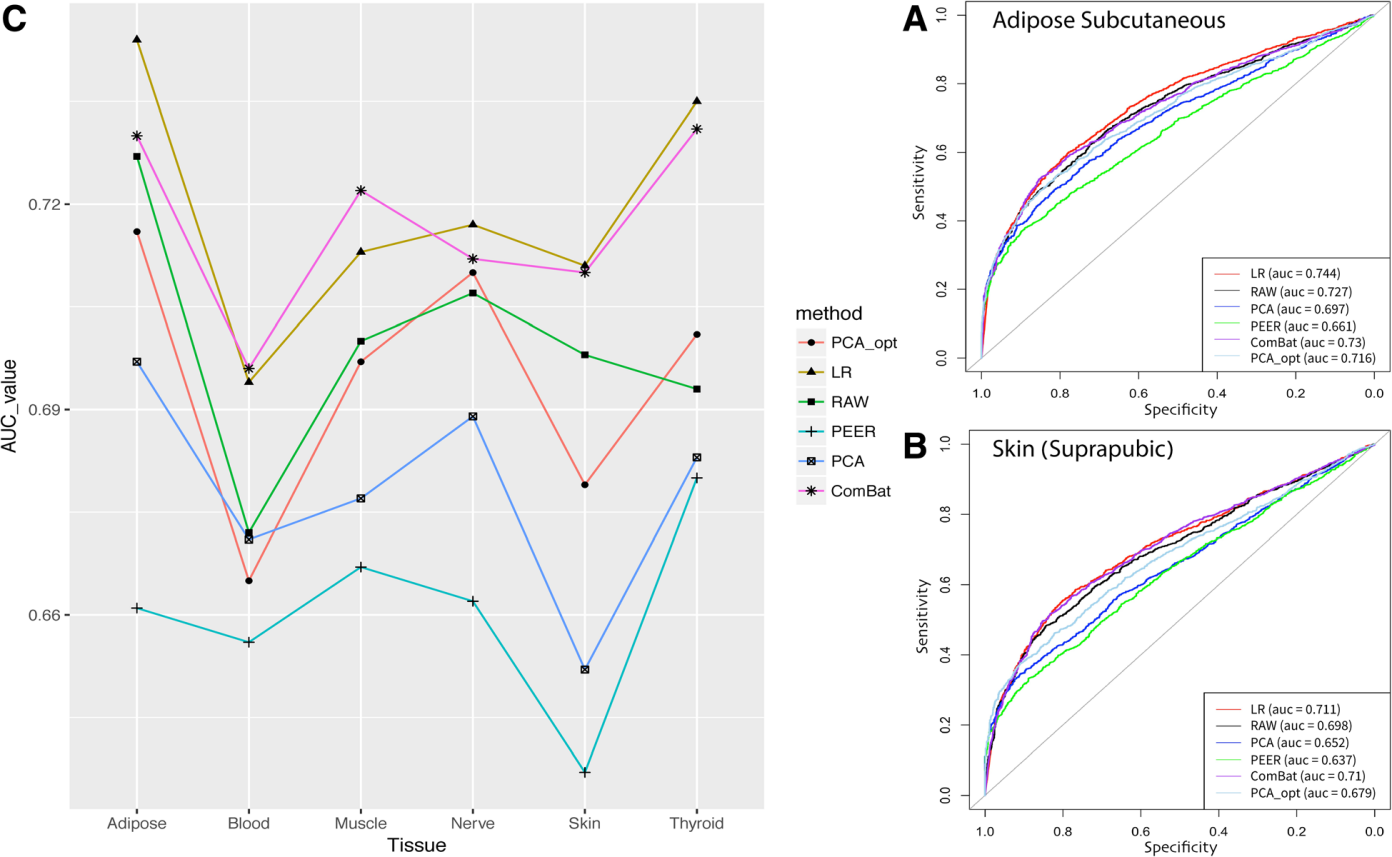
Performance evaluation of five adjustment methods and the raw data applied to six representative tissues. ROC curves and their corresponding AUC values are presented. ROC curves [30] are graphical representations of both specificity and sensitivity that take into account both the gene-gene co-expression of the adjusted dataset against the gold standard, a-priori knowledge of *true* and *false* gene-gene associations derived from the GIANT project [18]. (**a**) Performance evaluation for the Adipose Subcutaneous tissue dataset. Performance was evaluated using 2975 gold standard edges (1796 and 1179 *true* and *false* edges respectively) for this tissue. (**b**) Performance evaluation for the Skin - Not Sun Exposed (Suprapubic) dataset. Performance was evaluated using 2986 gold standard edges (1820 *true* and 1166 *false* edges). (**c**) Plot summarizing the AUC values for six tissue datasets (x-axis) and five adjustment methods and raw data (see Methods section). It can be seen that LR (linear regression-based adjustment for known confounders) and ComBat [4] outperforms the other adjustment methods

Figure 5 exemplifies the third step of our framework that includes the performance evaluation of five adjustment methods and raw data demonstrated for six tissues. Figure 5 a, b demonstrates the performance evaluation plot and AUC values after adjusting the “Adipose subcutaneous” and “Skin - Not Sun Exposed (Suprapubic)” tissue datasets with five batch correction methods and the raw unadjusted data. See Additional file 1: Figure S6 for performance evaluation plots of Whole-blood, Thyroid, Muscle Skeletal and Nerve Tibial tissue datasets. Figure 5c summarizes the AUC results of these six adjusted datasets for six different tissues. As expected, the more delicate “PCA_opt” adjustment (see Methods), which includes optimal principal components to be used as suggested by the method in [14], outperforms the “PCA_all” adjustment in most exemplified tissues. It can be seen that using the linear regression model and ComBat, which adjust for known confounders, outperform other methods, the PCA-based and factor analysis-based using PEER hidden covariates.

## Discussion

We present B-CeF, a new methodology for estimating the effectiveness and quality of gene expression data adjustment methods to preserve the genuine biological signal in actual data. The novelty of our approach is in using an a-priori high confident gene-gene co-association score based on real observations to evaluate adjustment methods. The a-priori knowledge of gene-gene associations is derived from the GIANT project [18] and calculated using thousands of gene expression and protein interaction experiments. As opposed to other approaches [13, 14] that assumed the existence of a co-expression network between the genes within a GO (Gene Ontology) category, our approach uses actual networks observed experimentally to be co-expressed and co-associated based on thousands of experiments. We complement the methodology with an R software package that can be easily downloaded and executed.

Choosing the adjustment approach for a highly heterogeneous dataset, such as GTEx, may be counterintuitive. Commonly, hidden confounders were inferred and used (e.g., [14] for co-expression analysis, [16] for eQTL analysis) to adjust the GTEx dataset, in order to cope with data heterogeneity. We show here, using our new framework, that linear regression-based methods and ComBat [4], adjusting for known confounders, outperform the methods adjusting for hidden confounders that remove some of the desired biological signal along with removing the data variability. Supporting our results, Mostafavi et al. [13] used GO (Gene Ontology) categories to show that unguided removal of top principal components significantly reduces the accuracy of co-expression networks compared to the raw RPKM data. Following this trend, the work in [14] optimized the number of principal components used (utilizing GO categories) to adjust the GTEx data for generating co-expression networks.

An important aspect of the approach is to correctly select the a-priori knowledge that is used. Choosing gene-gene co-association scores especially suited to the study at hand may improve the effectiveness of the approach. In our exemplified GTEx datasets, most of the gene expression profiles belong to healthy yet post-mortem donors, while the GIANT co-association scores are based on various types of phenotypes, e.g., diseased and healthy individuals. A future enhancement may be to generate dedicated gold standards, e.g., tissue-specific post-mortem healthy individuals that match the exact data set at hand. To overcome this limitation, we limited our analysis to the most confident gene-gene associations from GIANT [18] (we used the weakest and strongest edges derived from the tissue-naïve network, trained on all tissue types and conditions). These *true* and *false* gene-gene associations are verified to be co-associated across multiple biological conditions, which presents a strong basis for our framework. We note that the low confidence interactions (*false* gene-gene associations) may still have evidence in some specific tissue or condition, which may affect the performance scores and our results.

Nevertheless, publically available databases of highly confident co-expression networks based on thousands of experiments is in a grow. For example, the tissue-naïve and tissue-specific networks of the GIANT project [18], Gene Network [21] and the species-specific GeneFriends [22] currently include co-expression maps for human and mouse. These high confident network databases can serve as a basis for generating co-expression networks gold standards to be used by our framework.

The simplicity of B-CeF makes it flexible and an excellent tool for additional purposes. For example, it suits any gene expression experimental platform and can be used to infer the optimal number of principal components for adjusting the data with minimal effect on the expected biological signal.

## Conclusions

We show that using inferred hidden confounders that remove data variability overcorrects the data and results in a loss of essential biological signals. Our developed framework provides for evaluating the efficiency of batch correction methods in preserving original biological signals and can be used with any type of gene expression profile generated for any experimental platform.

## Methods

### A-priori co-expression network

The GIANT project [18] generated genome-wide functional interaction networks for 144 human tissues and cell types developed using a data-driven Bayesian methodology that integrates thousands of experiments (> 14,000 distinct publications) to yield a confidence score for each gene-gene interaction. The experiments were derived from GEO (Gene Expression Omnibus [23]) human datasets and biological interaction databases such as BioGRID [24]. We downloaded the tissue-naïve network gold standard (“all_tissue” full network from http://giant.princeton.edu/download/), which trained a classifier based on genome-wide functional interactions. The confidence score of a gene-gene association represents the probability for two genes to be associated over the multiple tissues/cell-types included in the project. We derived the first 100,000 gene-gene associations and their confidence scores from this network. We then extracted the highest/lowest confidence gene-gene pairs to represent true/false pairs. We define t*rue* edges as those having confidence > 0.5 and were assigned with the value 1, and *false* edges with confidence < 0.025 and were assigned with the value 0. We calibrated the confidence cutoffs (confidence scores are in the [0,1] interval) to balance between the number of the *true* and *false* associations. The calibration included initiating the low cutoff for a confidence of a *false* gene-gene association to 0.01 and the high cutoff for the confidence of a *true* gene-gene association to 0.7, and then increasing/decreasing the confidence cutoffs by 0.005/0.05 respectively until the number of *true* and *false* associations were approximately balanced. The final set of *true* and *false* associations includes 3490 associations (1935 *true* associations and 1555 *false* associations) used as the gold standard for the performance calculations. The number of actual gold standard associations used per tissue was slightly lower since we removed associations between tissue-specific low expressed genes. Finally, we used the following number of edges: (1) Adipose Subcutaneous - 1796 *true* and 1179 *false* edges, (2) Skin - Not Sun Exposed (Suprapubic) - 1796 *true* and 1179 *false* edges, (3) Muscle – Skeletal - 1736 *true* and 1062 *false* edges, (4) Nerve - Tibial - 1789 *true* and 1120 *false* edges, (5) Thyroid - 1809 *true* and 1176 *false* edges and (6) Whole Blood - 1770 *true* and 1063 *false* edges.

### GTEx data set

We applied our approach to six representative tissue expression profiles derived from the Genotype Tissue Expression Project (GTEx) [15, 16]. GTEx is a large-scale heterogeneous human tissues dataset of RNA-seq data, e.g., it contains 298 adipose subcutaneous samples and 196 skin (not sun exposed from the suprapubic) samples. We downloaded the gene expression tissue-specific datasets [25] (version V6) from the GTEx portal. The downloaded data included pre-processed RPKM values, along with a phenotype matrix and per-tissue PEER inferred covariates files (e.g., Adipose_Subcutaneous_Analysis.v6p.covariates.txt file). The pre-process of these datasets included [16] (1) filtering for average gene expression > 0.1 RPKM and RIN (RNA Integrity Number) values greater than 6, (2) quantile normalization within each tissue and (3) mapping each gene set of expression values to a standard normal distribution. The per-tissue 15 PEER factors were generated [16] using the top 10,000 expressed genes per tissue and normalized with the same procedure as described for the expression matrices.

### Data correction

We evaluated five methods that correct for known and hidden confounders.

### The following correction methods were tested

1. *LR (Linear Regression):* the multiple linear regression model was used to fit for gender (GENDER), ischemic time (SMTSISCH representing the interval in minutes between time of donor death and sample collection), age (AGE), experimental batch (SMGEBTCH) and death type (DTHHRDY) for the gene expression data. We derived the relevant phenotype vectors from the downloaded phenotype table.
2. *PEER:* We used 15 inferred PEER factors (see GTEx dataset description above) and gender to adjust for the data. PEER factors are hidden covariates inferred using a factor analysis-based approach [6].
3. *PCA*: the principal components that accounted for most of the variability in the data set (9, 10, 10, 9, 10, 10 first principal components for adipose subcutaneous, skin, nerve, muscle whole blood and thyroid respectively, see Additional file 1: Figure S1) and gender were used to adjust the data.
4. *PCA_opt*: same as PCA but adjusted for optimal number of principle components as reported by [14] (5, 5, 4, 4, 7 principal components for adipose subcutaneous, skin, nerve, muscle and whole blood respectively).
5. *ComBat*: We executed ComBat [4] using the ‘sva’ R package [7] to adjust for death type, experimental batch, ischemic time, age and gender. Due to the discrete nature of ComBat, the continuous ischemic time values were discretized into five bins, labels 1 to 5, by partitioning them into 300 min intervals. Age includes the 20–80 year range and is partitioned into 10 year intervals (embedded in the GTEx dataset). We removed genes with zero variance per each batch group and type. We removed batches with one sample within a batch. Since ComBat [4] is not designed to correct for multiple batch effects simultaneously, we adjusted each batch iteratively, accounting for the yet unadjusted batches in each iteration.

We tested a sixth method that uses singular value decomposition and a permutation test for choosing the number of singular vectors to be included in the adjustment. It showed similar trend as the PCA-based adjustment (see Additional file 1: Figure S7 in the supplemental file for results and method explanation).

For batch correction methods 1–4 above (i.e., except for ComBat, which generates the adjusted dataset), we used the multiple linear regression model to extract the gene expression residual of gene *i* in sample *j* computed as follows:

$$ \operatorname{Re}{sidual}_{\mathrm{i}}^{\mathrm{j}}={Exp}_{\mathrm{i}}^{\mathrm{j}}-\sum \limits_{n=1}^N{Coef}_{i,n}\;{Confounder}_{\mathrm{n}}^{\mathrm{j}} $$

*Exp*_i_^j^ is the expression level of gene *i* in sample *j*, *Confounder*_n_^j^ is the n-th confounder (can represent a principal component, PEER factor or known covariates) in sample *j*, N is the number of confounders considered, *Coef*_*i*,n_ is the regression coefficient of gene *i* on confounder n. The residuals from the regression calculation were treated as the expression level of each gene. We used the R ‘stats’ package to generate the computations.

### Gene-gene association measure

We measured gene-gene pair co-associations using the Spearman correlation coefficient or Spearman’s rho [26]. Spearman correlation is a nonparametric rank-based correlation calculation method that provides a robust measure of a nonlinear monotonic relationship between two continuous or discrete ordinal variables not enforcing a bivariate normal distribution on the variables. The method uses linear relations between the ranks of the values of the two variables and is generally more robust to outliers. Spearman correlation uses the same formula as the Pearson correlation [26] except that the values of the variables are replaced with their ranks. In case of tied (equal) values, they are assigned a rank that is the average of their positions in the ascending order of the values. Mathematically, for a sample size n, the raw values *x*_*i*_, *y*_*i*_ are converted to their corresponding ranks *x*_*i*_^*rank*^, *y*_*i*_^*rank*^ and the Spearman correlation coefficient *r*_*s*_ is computed as follows:

$$ {r}_s=\frac{\operatorname{cov}\left({x}_i^{rank},{y}_i^{rank}\right)}{\sigma_{x_i^{rank}}\;{\sigma}_{y_i^{rank}}} $$

$\mathit{\operatorname{cov}}\left({x}_i^{rank},{y}_i^{rank}\right)$ is the covariance of the rank variables and ${\sigma}_{x_i^{rank}},{\sigma}_{y_i^{rank}}$ are the standard deviations of the rank variables *x*_*i*_^*rank*^, *y*_*i*_^*rank*^ respectively. If all n ranks are distinct integers (i.e., not tied), the Spearman correlation coefficient can be computed using the formula:

$$ {r}_s=1-\frac{6{\sum}_{i=1}^n\left({d}_i^2\right)}{n\left({n}^2-1\right)} $$

Where n is the number of observations and *d*_*i*_ = *x*_*i*_^*rank*^- *y*_*i*_^*rank*^ is the delta between the two ranks of each observation. *r*_*s*_ is a measure between − 1 and 1 (representing perfect negative/positive correlation respectively). The Spearman’s rho calculation is specifically appropriate for identifying gene expression values that are co-elevated and co-decreased in a monotonic manner, and in a comparative study it was found to perform better for constructing a gene co-expression network [27]. We computed the *p*-values of the correlation using the student’s t distribution approximation [28], where t has a student t-distribution with n-2 degrees of freedom. We used the cor.test R function from the stats package for the calculations.

### Effectiveness evaluation of adjustment methods

For each gold standard *true* and *false* gene-gene pair, we generated the corresponding Spearman correlation coefficients and *p*-values for the “raw” unadjusted dataset and the five adjusted datasets (the raw dataset adjusted with five methods). We excluded pairs where at least one of the genes was absent from the tissue-specific GTEx datasets (e.g., filtered since low expression). We scored each pair using the following metric: –log10(adjusted p-values(r_s_(g_1_, g_2_)), where g_1_ and g_2_ represent the expression of each two genes consisted in a gene-gene pair derived from the gold standard and r_s_ their Spearman correlation coefficient estimate. The p-values were adjusted for multiple comparisons using BH (Benjamini-Hochberg correction) [29].

We chose ROC curves [30] and AUC measures [31] to assess the performance in our framework. The receiver operator characteristic (ROC) curve [30] is a commonly used standard measure to evaluate classification performance. ROC curves [30] evaluate the performance of each method by plotting the true positive rate (i.e., sensitivity) against the false positive rate (i.e., 1-specificity) at various threshold settings. The actual test statistic is the area under the curve (AUC), and the dataset with the optimal combination of sensitivity and specificity will have the largest area of AUC [31]. There are other measures of classification accuracy, e.g., Brier score [32] or precision-recall curves [33]. Precision-Recall (PR) curves may give a more informative picture of an algorithm’s performance when dealing with highly skewed datasets [33]. Hanczar et al. [34] compared performance measurements on simulations at various sample sizes up to 1000 observations and detected AUC inaccuracies in imbalanced samples and smaller samples. Taking these into account, AUC measurement is optimal for large-scale sample size and balanced sample distribution. We balanced our class distribution (the *true* and *false* edges) and our sample size to includes > 3000 samples, which makes ROC curves analysis highly suitable for assessing the effectiveness of each adjustment method in our framework.

We generated ROC-AUC for GTEx RPKM raw data and the five adjustments. The method that performs better (higher AUC) than others is suggested to be more effective. The evaluation of overall performance was executed using the R ‘pROC’ package.

## Additional file

Additional file 1:  Analysis of explained variability and performance evaluation of adjustment methods in several tissues. (DOCX 1334 kb)

## Abbreviations

AUC: Area Under the Curve
eQTL: expression Quantitative Trait Loci
GO: Gene Ontology
ROC: Receiver Operator Characteristic
RPKM: Reads Per Kilobase Million
