# Supplementary material for: Batch correction evaluation framework using a-priori gene-gene associations: applied to the GTEx dataset
Source: BMC Bioinformatics. 2019 May 28;20:268. doi: 10.1186/s12859-019-2855-9 (PMC6537327; doi:10.1186/s12859-019-2855-9)
Supplement: Supplementary file 1 — Analysis of explained variability and performance evaluation of adjustment methods in several tissues. (DOCX 1334 kb) [file 12859_2019_2855_MOESM1_ESM.docx]

# Supplemental File:

# Batch correction evaluation framework using *a-priori* gene-gene associations: applied to the GTEx dataset

Judith Somekh^a, b, c^, Shai S. Shen-Orr^b^, Isaac Kohane^a^

^a^ Department of Biomedical Informatics, Harvard Medical School, Boston, MA, USA

^b^ Faculty of Medicine, Technion – Israel Institute of Technology, Haifa, Israel

^c^ Department of Information Systems, University of Haifa, Haifa, Israel

*
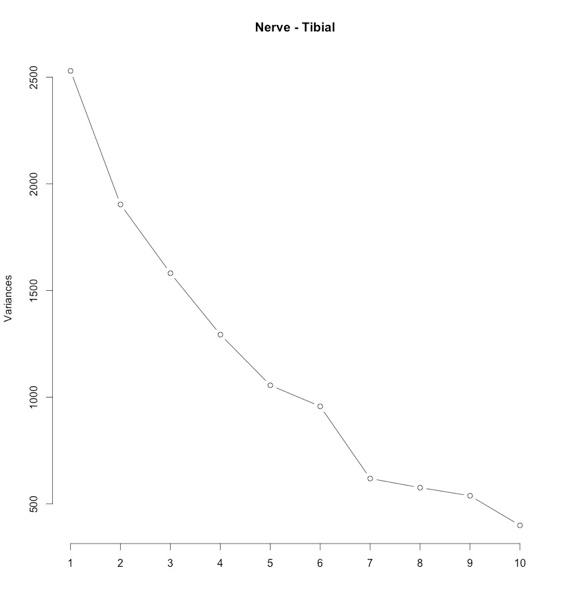

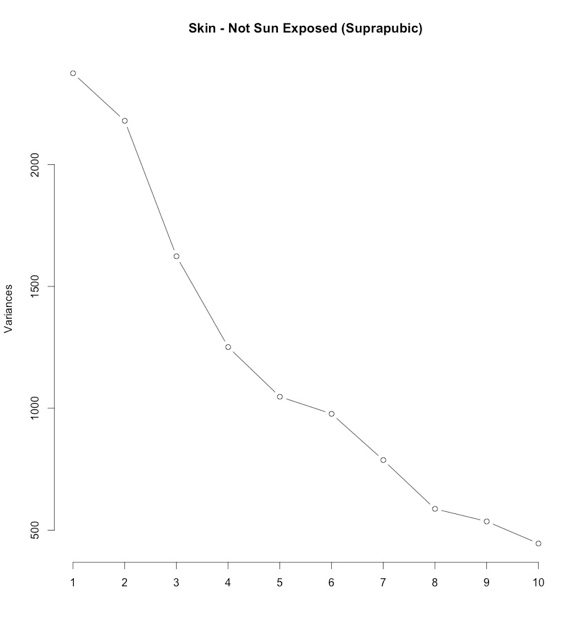
*

*
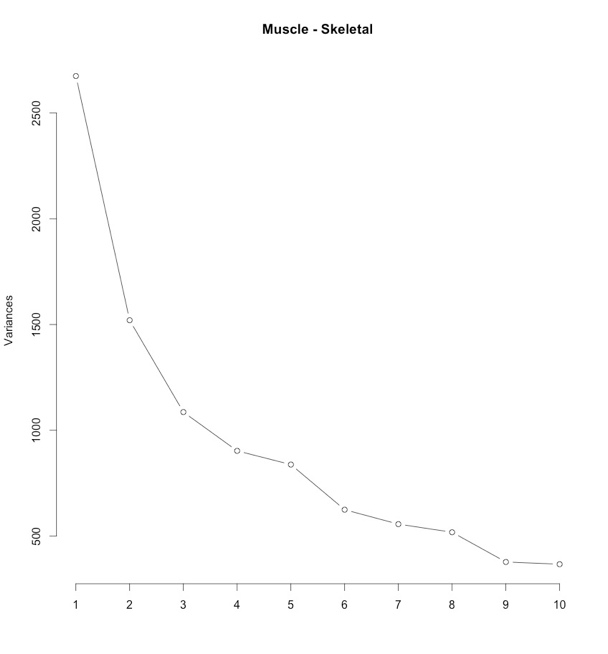

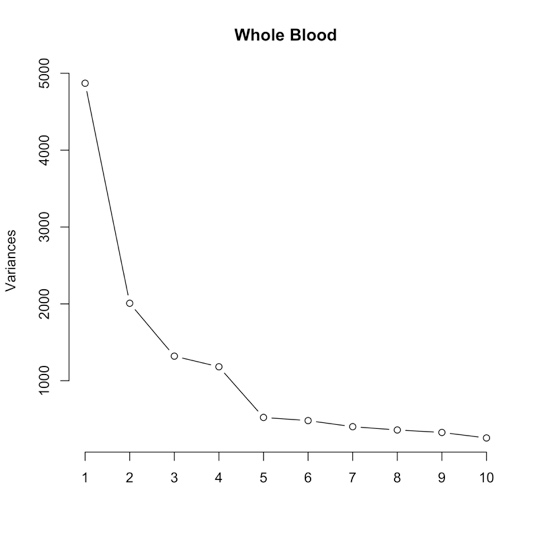
*

Figure S1. The x-axis represents the number of principle components used and the y-axis the effect on variance.


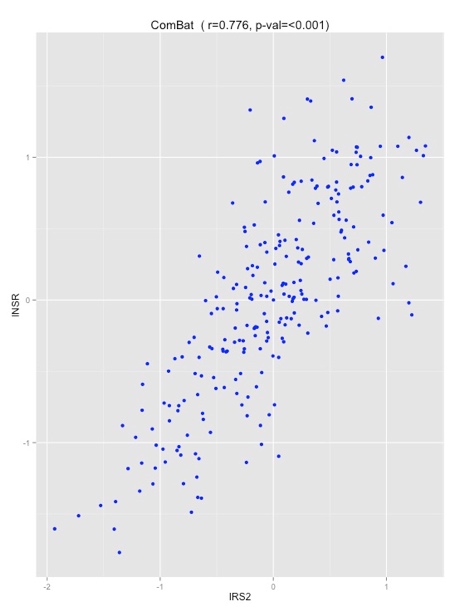

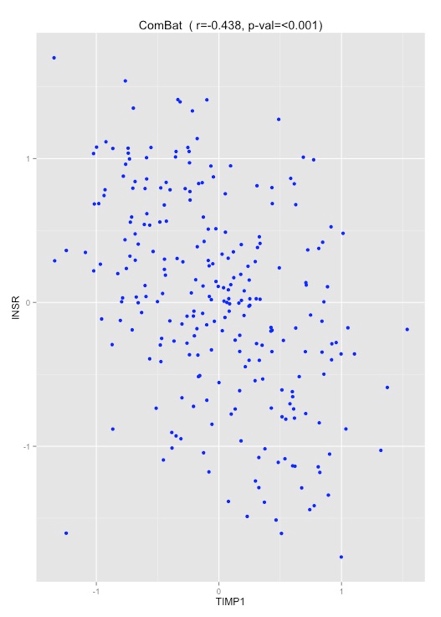
*
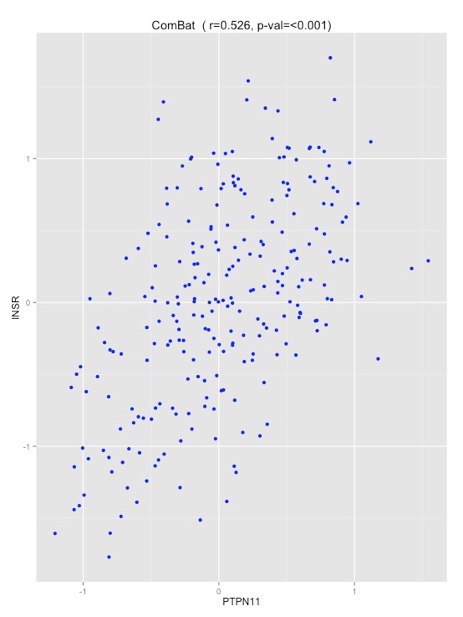
*

Figure S2. Correlation coefficients of three true gene-gene associations after adjustment with Combat for the GTEx Adipose Subcutaneous dataset.

Figure S3. Comparison of Correlation coefficients of three true gene-gene associations after adjustment with LR, PCA-based and Combat.

1. (B)

Figure S4. True positives (%) for each adjustment method for a predefined cutoff, calculated for the Adipose Subcutaneous tissue dataset. (A) True positives (%) for true strong confidence edges (correlation coefficient cutoff is >0.4 or <-0.4) (B) True positives (%) for false low confidence edges (correlation coefficient cutoff is in the interval [-0.1, 0.1]). It can be seen from (A) that methods reducing most data variability detects only 4% of the true edges.

A2

A1

B1

B2

C2

C1

Figure S5. Density plots of correlation coefficients of gene-gene pairs following adjustment with five methods and raw data ("RAW"). Muscle - Skeletal dataset: (A1) Density plot for false gene-gene pairs. (A2) Density plot for true gene-gene pairs. Nerve - Tibial dataset: (B1) Density plot for false gene-gene pairs. (B2) Density plot for true gene-gene pairs. Skin – Not Sun Exposed dataset: (C1) Density plot for false gene-gene pairs. (C2) Density plot for true gene-gene pairs. Data adjustment reducing most variability with hidden confounders, i.e., PEER and PCA-based covariates (colored in green and blue respectively) demonstrates a tendency toward zero mean of the correlation coefficients both in the false and in the true gene-gene pairs.


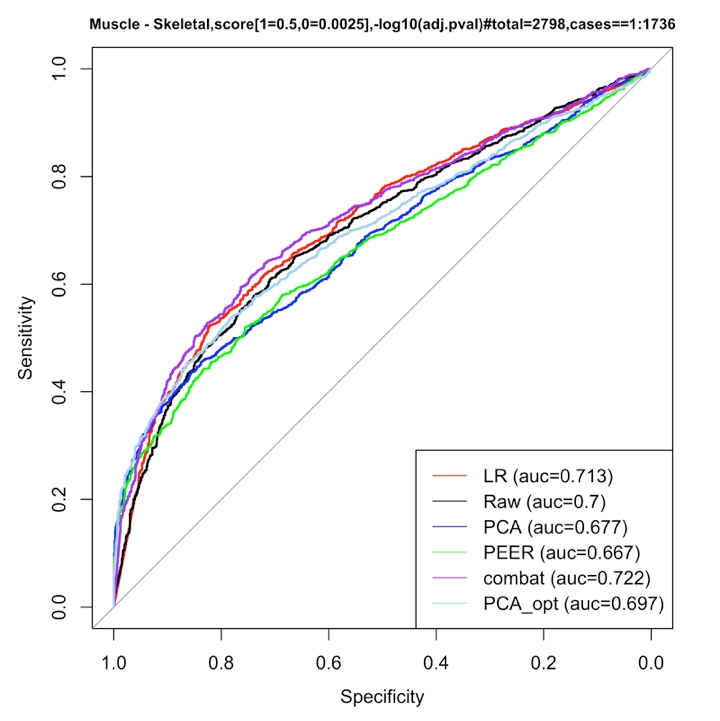

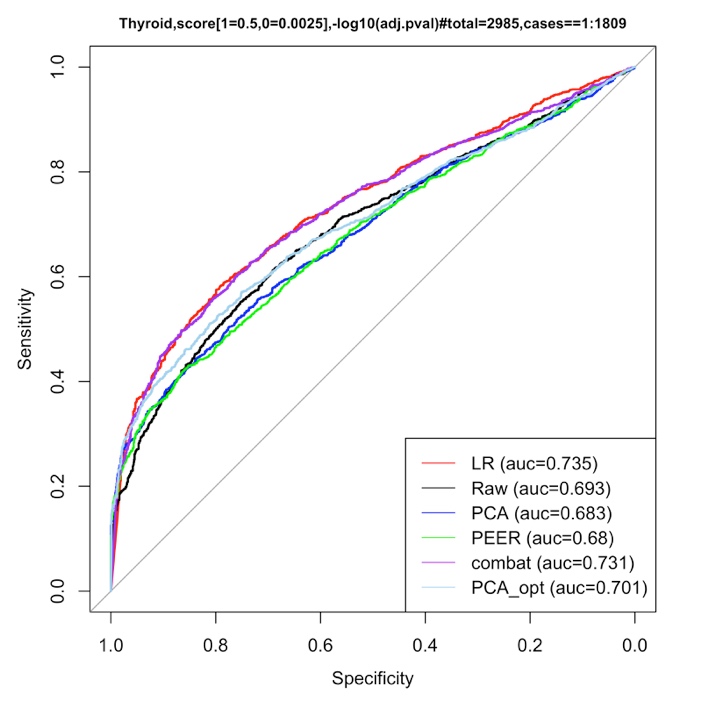

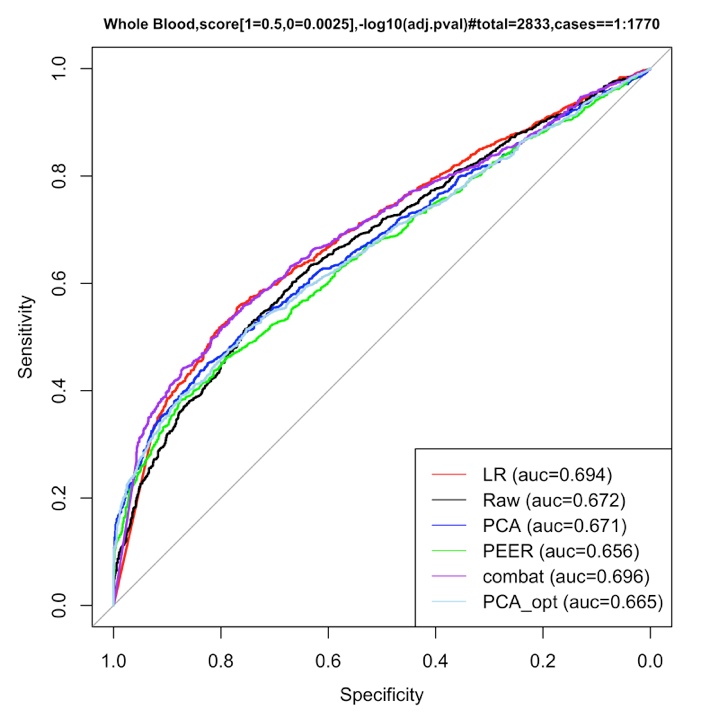

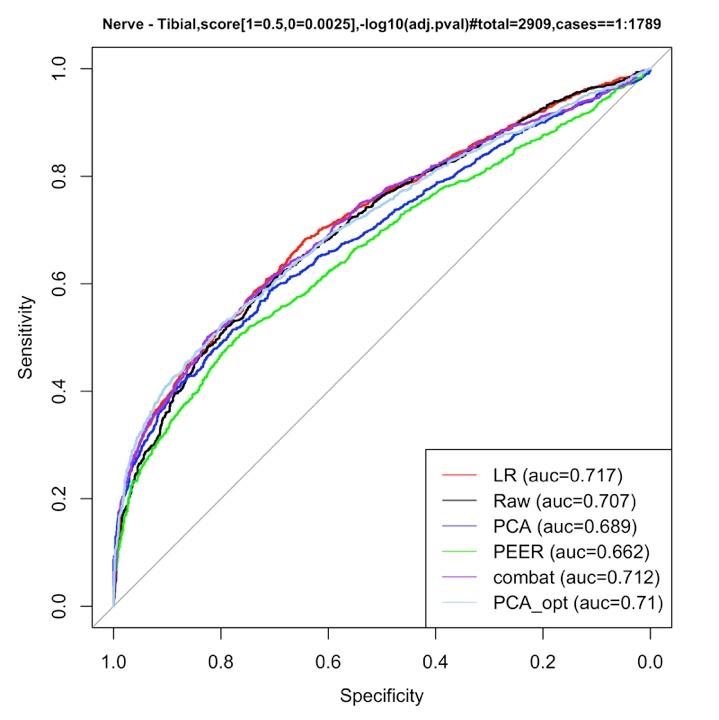


B

A

C

D

Figure S6. Performance evaluation, ROC curves and AUC, of five batch correction methods and raw data (RAW), applied to four representative tissue datasets derived from the GTEX project. ROC curves are graphical representations of both specificity and sensitivity that considers both the gene-gene co-expression of the tested dataset against the gold standard a-priori knowledge of true and false gene-gene associations derived from the GIANT project. (A) Muscle Skeletal tissue data set. (B) Thyroid tissue data set. (C) Whole blood tissue data set. (D) Nerve tibial tissue data set.


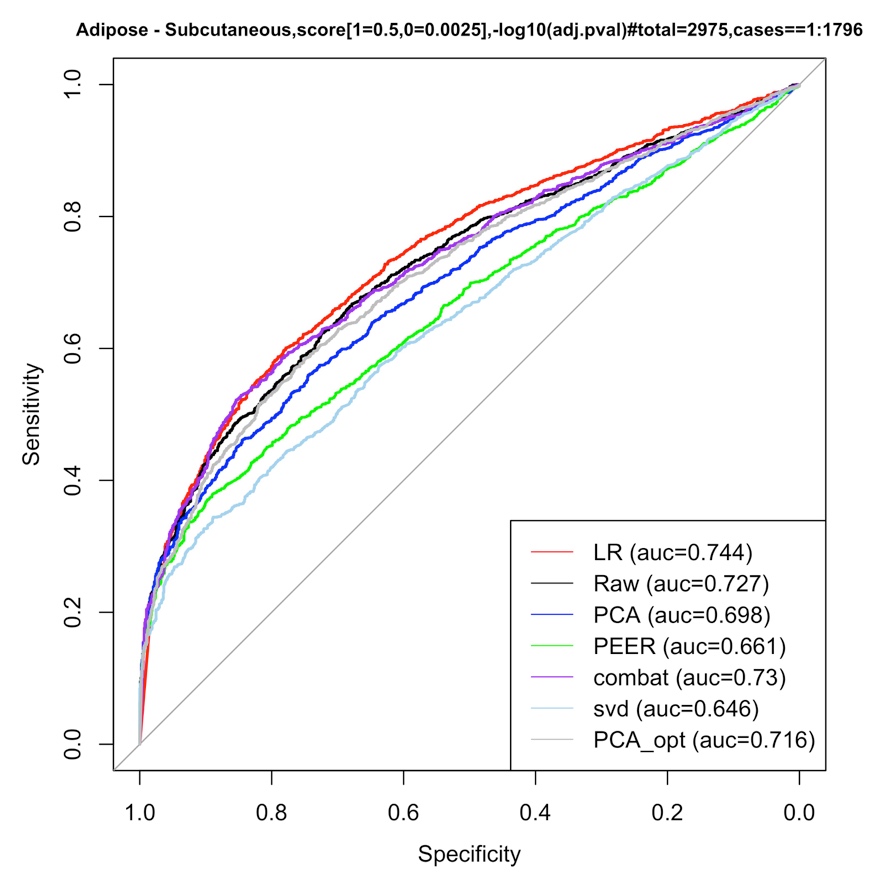


Adipose Subcutaneous

Figure S7. Performance evaluation, ROC curves and AUC, of six batch correction methods and raw data (RAW), applied to Adipose Subcutaneous tissue datasets derived from the GTEX project. Here we additionally assessed the "svd" approach, which shows the same trend. The "svd" approach uses singular value decomposition to compute singular vectors from the expression dataset and estimates the number of singular vectors to be used in the adjustment by conduction a permutation test, as suggested in [‎1]. 34 significant singular values were used to adjust the dataset. The "svd" R function and "num.sv" function from the "sva" Bioconductor R package [‎2] were utilized for the calculation"

**References**

1. Buja A, Eyuboglu N. Remarks on Parallel Analysis. Multivariate Behav Res. 1992;27:509–40.
2. Leek JT, Johnson WE, Parker HS, Jaffe AE, Storey JD. The sva package for removing batch effects and other unwanted variation in high-throughput experiments. Bioinformatics. 2012;28:882–3.
